# Computer Vision-Based Airport Turnaround Monitoring Using YOLOv11, Multi-Object Tracking, and Motion-Based Passenger and Baggage Activity Detection

**DOI:** 10.3390/s26134231

**Published:** 2026-07-03

**Authors:** Nutchanon Suvittawat, De Wen Soh

**Affiliations:** Information Systems Technology and Design, Singapore University of Technology and Design, Singapore 487372, Singapore; nutchanon_suvittawat@mymail.sutd.edu.sg

**Keywords:** airport turnaround, computer vision, YOLOv11, object detection, Norfair tracking, frame differencing, passenger detection, baggage detection, ground support equipment, Gantt chart, airport operations

## Abstract

Airport turnaround is an important operational process that directly affects flight punctuality, airport capacity, and ground-handling efficiency. However, many turnaround activities are still monitored manually or through fragmented operational records, which can limit real-time visibility and delay identification. This study proposes a computer vision-based airport turnaround monitoring pipeline that integrates YOLOv11 object detection, Norfair multi-object tracking, and frame differencing-based motion analysis to extract key operational events from airport video footage. Publicly available turnaround footage from Shinshu Matsumoto Airport, Japan, was collected under different environmental conditions, including daytime, nighttime, rainy, after-rain, and transition lighting conditions. From selected videos, 1446 images were labeled into 11 airport turnaround object classes, including tow tug, aerobridge, airplane, baggage container, belt loader, belt loader roof, fuel line, fuel tanker, fuel tube, tractor, and window. The dataset was divided into training, validation, and testing sets using a 70:20:10 ratio. The trained YOLOv11 model achieved strong detection performance, with overall test an precision of 0.9609, recall of 0.9445, and mAP50 of 0.9617. To support activity-level interpretation beyond object detection, the proposed pipeline applies frame differencing within specific regions of interest, including the aerobridge window region for passenger deboarding and boarding detection, and the belt loader roof region for baggage unloading and loading detection. The extracted object detections, motion spikes, and temporal logs are then converted into a Gantt chart that summarizes major turnaround activities, including airplane parking, deboarding, baggage unloading, refueling, baggage loading, boarding, and pushback. The results demonstrate that the proposed modified YOLO-based pipeline can transform ordinary airport video footage into structured operational timelines, supporting more transparent, data-driven, and automated monitoring of airport turnaround processes.

## 1. Introduction

**Figure 1:**
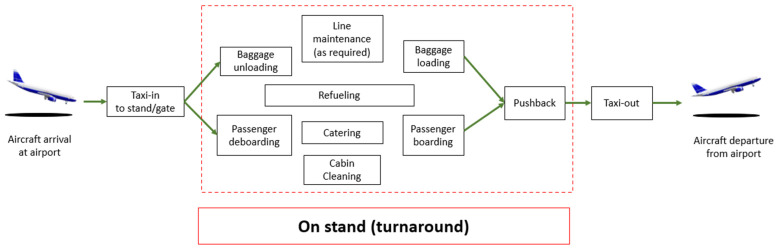
Airport turnaround process diagram.

Airport turnaround—the set of ground operations conducted between an aircraft’s arrival and its subsequent departure—is a critical determinant of on-time performance and operational efficiency. After parking at the assigned stand, multiple coordinated activities occur in parallel: the passenger aerobridge docks; passengers deboard; ground crews unload baggage using belt loaders and tractors to transfer containers to the terminal; refueling is performed via hydrant systems or fuel trucks; and, when required, catering is replenished. Cabin cleaning and light line maintenance (e.g., routine A-checks) are often executed within this window to ensure safety and service readiness. Once loading and boarding are complete and all ground equipment is clear, the pushback tractor positions the aircraft for taxi and departure [1,2,3]. Figure 1 summarizes these stages within a standard turnaround flow.

The rebound in air travel demand following the COVID-19 pandemic [4,5,6] has intensified pressure on airport infrastructure and staffing. Many airports still rely heavily on human observation and ad hoc documentation to monitor turnaround progress, diagnose delays, and coordinate resources. Scaling this manual approach typically requires hiring additional personnel, which increases costs and can introduce variability, delayed situation awareness, and avoidable disruptions [1,7,8].

This paper proposes an AI-enabled monitoring framework that integrates computer vision with existing terminal CCTV/webcam feeds to automatically track the status of key turnaround activities from arrival to pushback. The system infers event start–end times and generates a Gantt-style timeline with timestamps and durations for each task (e.g., deboarding, baggage unloading/loading, refueling, cleaning, boarding). By transforming raw video into structured operational telemetry and visual summaries, the approach supports early detection of delays and anomalies, reduces the need for manual time-and-motion logging, and provides controllers and ramp managers with a concise, real-time picture of turnaround health.

Our contributions are threefold: (i) a modular computer vision pipeline tailored to the airport ramp context that detects and associates ground assets, passenger flows, and aircraft–equipment interactions; (ii) a robust event-parsing method that converts detections into task timelines suitable for supervisory decision-making; and (iii) a practical visualization layer that renders per-flight Gantt charts for post hoc analysis and live operations. Collectively, these elements aim to improve turnaround transparency, support staffing efficiency, and help airports meet rising demand without compromising safety or service quality.

## 2. Research and Industry Survey

There are some research papers and commercial work that proposed various methods using artificial intelligence to help airport management to handle the flow of airplanes and passengers in part of the turnaround process as can be seen in Table 1 below.

**Figure 2:**
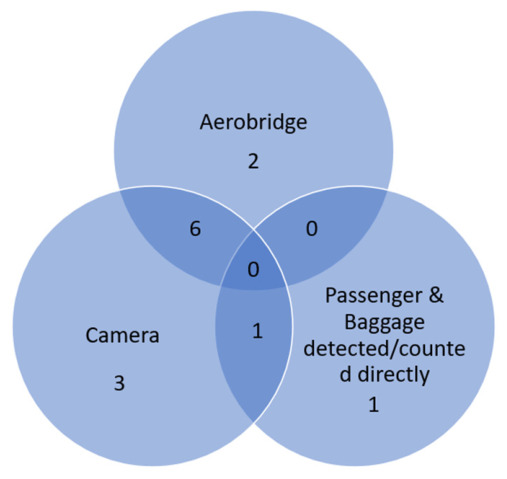
A Venn diagram showing gaps concluded from the literature review table.

Furthermore, the reviewed studies can be categorized according to the type of passenger access considered. Six studies focused exclusively on aerobridge-based operations [12,13,14,15,20,21,23], noting that [13,14] refer to the same work. Two studies considered only passenger stairs or ladders [17,19], while two studies addressed both aerobridge- and stair-based access [9,22]. Therefore, in the body of literature summarized in Figure 2, several important gaps can be identified. Most notably, existing computer vision studies on airport turnaround monitoring largely emphasize the detection of the aerobridge and other large ground support equipment to infer operational phases, while limited attention has been given to the direct detection of passengers and baggage within the same field of view, particularly when the aerobridge is simultaneously present in the camera frame. As a result, current approaches often provide only a coarse representation of turnaround activities and may not capture fine-grained operational transitions.

This limitation is important because passengers and baggage are small, dynamic objects whose movements are directly associated with critical turnaround sub-processes, including deboarding, boarding, baggage unloading, and baggage loading. Without explicitly detecting these objects, it is difficult to determine the exact start and end times of these activities with high precision. Addressing this gap would enable more accurate event-level timestamping and duration estimation, thereby improving the realism and operational validity of airport turnaround monitoring. Such fine-grained detection capability could also enhance the usefulness of computer vision systems for performance analysis, process optimization, and decision support in airport ground operations.

Additionally, two recent YOLOv5-based airport turnaround studies are particularly related to the present work. The authors of [17] proposed a vision-based framework for automatically collecting key milestone nodes, including in-block/off-block and docking/undocking events. The authors of [20] developed an improved YOLOv5-based apron surveillance method by introducing an SPD-Conv block to improve small-object detection for ground service object and activity recognition. These studies demonstrate the usefulness of YOLO-based perception for airport apron and turnaround monitoring.

However, the present study differs from these works in its monitoring objective and output structure. Instead of focusing mainly on selected key milestone nodes or improving the YOLO architecture itself, this work uses YOLOv11 as the object detection module within a broader turnaround monitoring pipeline. The proposed framework combines object detection, Norfair tracking, region-of-interest frame differencing, and rule-based temporal event parsing to estimate multiple turnaround activities, including airplane parking, passenger deboarding, baggage unloading, refueling, baggage loading, passenger boarding, and pushback.

Therefore, the main contribution of this study is not only object detection, but the transformation of detection, tracking, and motion information into structured operational monitoring outputs, including timestamped activity logs and a Gantt-style turnaround timeline.

## 3. Data Collection

**Figure 3:**
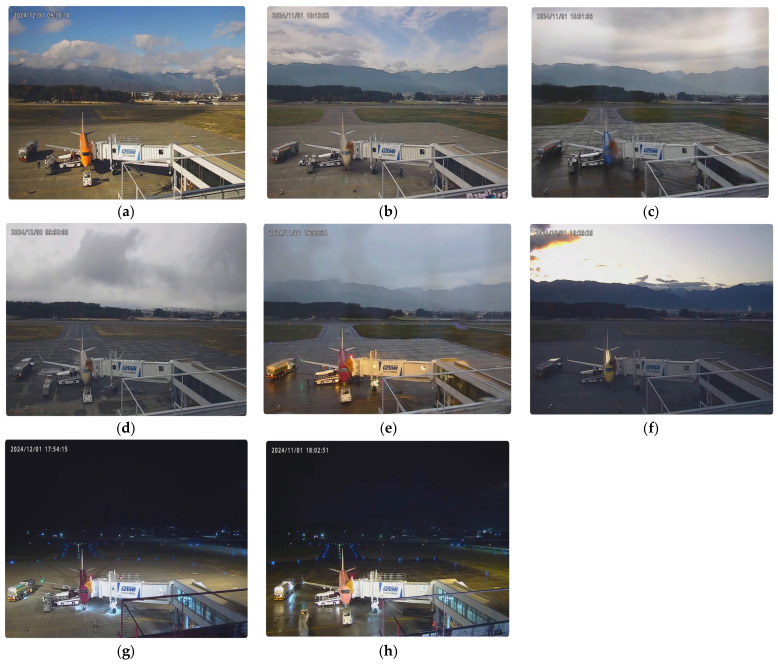
Image dataset environmental conditions that were used to train and test the AI model: (**a**) Daytime (sunny) without rain; (**b**) daytime (not sunny) without rain; (**c**) daytime with rain; (**d**) daytime after rain; (**e**) transition from daytime to nighttime with rain; (**f**) transition from daytime to nighttime without rain; (**g**) nighttime without rain; and (**h**) nighttime after rain.

**Figure 4:**
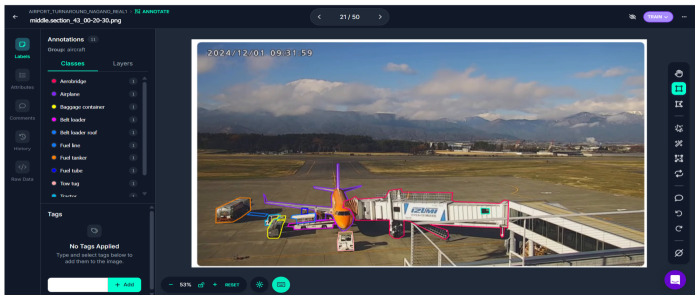
Example of an annotated airport turnaround image prepared in Roboflow 3.0. The image shows manually labeled bounding boxes for key turnaround objects, including the aircraft, aerobridge, belt loader, baggage container, fuel-related equipment, tractor, tow tug, and window.

In this research we collected airport turnaround footage of Shinshu Matsumoto Airport, Japan, from this public-accessed available website [25] from 16 October 2024 to 10 December 2024 with a video resolution 1920 x 1080 HD. And then video frames were extracted at 15 s intervals to construct the image dataset. This interval was selected to reduce redundancy compared with consecutive-frame extraction while still capturing changes in aircraft turnaround activities. Although frames extracted from the same video may share the same camera viewpoint, airport stand layout, aircraft position, and background features, they are not exact duplicates because passengers, vehicles, baggage containers, belt loaders, tractors, tow tugs, and other ground support objects may move between sampled frames. Nevertheless, because the dataset split was performed at the image frame level, visually related frames from the same video may appear across the training, validation, and test sets. This may lead to more optimistic object detection performance than a strict video-level split. Therefore, future work should evaluate the proposed method using video-level or airport-level splits to better assess generalization to unseen turnaround sequences, camera viewpoints, and airport layouts. The videos that we selected represented every possible environmental condition as much as possible, such as shown in Figure 3. After we screenshotted those eight selected turnaround videos, we had 1446 images in total for our dataset, then we used the Roboflow website [26,27] to label those objects that appear in the image scenes. The list of objects that we labeled are 1. tow tug, 2. aerobridge, 3. airplane, 4. baggage container, 5. belt loader, 6. belt loader roof, 7. fuel line, 8. fuel tanker, 9. fuel tube, 10. tractor, and 11. window. A labeled example image can be seen in Figure 3 and Figure 4.

**Figure 5:**
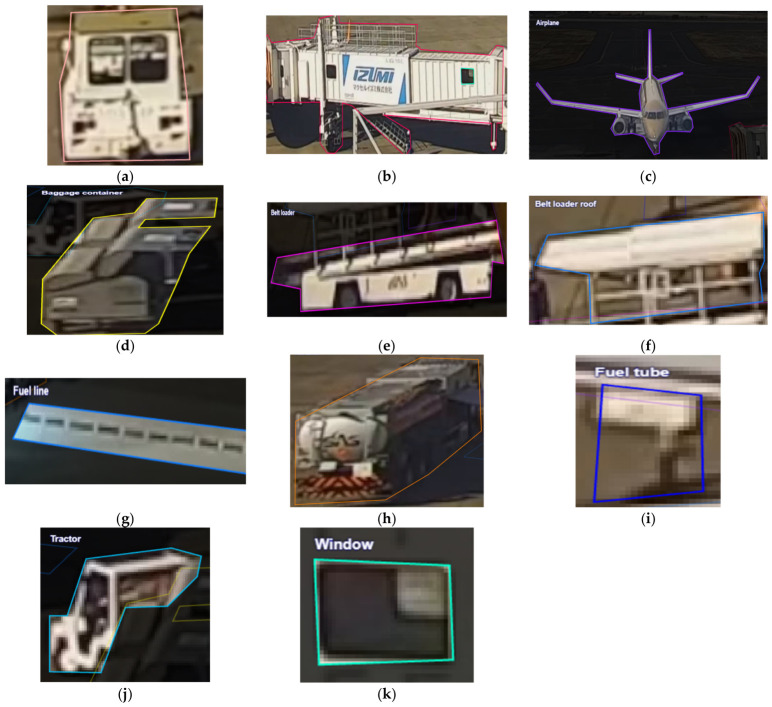
Example images of object by classes using Roboflow: (**a**) Tow tug, (**b**) aerobridge, (**c**) airplane, (**d**) baggage container, (**e**) belt loader, (**f**) belt loader roof, (**g**) fuel line, (**h**) fuel tanker, (**i**) fuel tube, (**j**) tractor, (**k**) window.

Then, after we finished labeling all of 1446 images, we randomly divided them into three sets using a 70:20:10 ratio [28,29]—1. Training set with 1012 images (70%), 2. validation set with 288 images (20%), and 3. test set with 146 images (10%)—as this is a good ratio for image processing in aviation-related topics, as shown in our previous work [30]. Then, every set was assigned every object class, as shown in Figure 5, with various number of instances shown in Table 2.

## 4. Methodology

### 4.1. Workflow of the Proposed Airport Turnaround Monitoring Pipeline

#### 4.1.1. Input Acquisition

The first stage of the workflow is the collection of airport turnaround video footage. This video serves as the raw observational data for the system. It contains visual information on aircraft position, ground support vehicles, aerobridge connection, passenger movement, and baggage-handling activities. The footage is then passed into the processing pipeline for automatic analysis.

#### 4.1.2. Pipeline Initialization

After the input video is loaded, the system initializes all major processing components. These include:

- The Ultralytics YOLO model with trained weights (best.pt) for object detection;
- The Norfair tracker for multi-object tracking across consecutive frames;
- A class color map for visual annotation;
- Supporting variables such as timers, logs, and temporary buffers for event recording.

This initialization stage ensures that the detection, tracking, visualization, and logging modules are ready before frame-by-frame analysis begins.

#### 4.1.3. Video Property Reading and Effective Frame Rate Computation

Next, the system reads essential video properties such as frame rate (fps) and frame size. Based on these properties, the pipeline computes the effective fps, particularly when frame skipping is applied to reduce computational cost. This step is important because all temporal measurements, such as event duration and timestamp generation, must be consistent with the actual number of processed frames per second.

#### 4.1.4. Object Detection Using YOLOv11

Once video properties are prepared, each frame is processed using YOLOv11 object detection. At this stage, the model identifies relevant airport turnaround objects, such as aircraft, aerobridge, belt loader, baggage tractor, baggage container, fuel equipment, and other operational elements visible in the scene.

The role of this stage is to convert raw image frames into structured detection outputs, including object class labels, bounding box locations, and confidence scores. These detections form the basis for the subsequent tracking and event analysis steps.

#### 4.1.5. Multi-Object Tracking Using Norfair

The detected objects are then passed to the Norfair tracking module. Tracking is used to maintain object identities across frames, allowing the system to determine how long each object remains visible and how its position changes over time.

This stage is necessary because airport turnaround analysis depends not only on object presence but also on temporal continuity and motion behavior. By assigning persistent IDs to detected objects, the system can support event interpretation, duration measurement, and more reliable logging.

#### 4.1.6. Branching into Two Analytical Pathways

After tracking, the workflow splits into two complementary analytical branches:

##### Event Logic from Spatial and Movement Relations

The first branch analyzes spatial relationships and motion interactions among detected and tracked objects. This branch is used to infer operational events that depend on the relative arrangement or coordinated movement of multiple objects. According to the diagram, this logic is used for events such as refueling, airplane parking, pushback.

##### Frame Differencing for Motion Spike Analysis

The second branch performs frame differencing, which is used to detect motion changes between consecutive frames. This technique highlights areas of activity by measuring pixel-level differences over time. In the workflow, frame differencing is specifically applied to detect motion spikes, which are useful for identifying activities that are difficult to represent reliably using bounding-box detection alone.

From this motion-based branch, two important airport turnaround processes are derived:

- Passenger deboarding/boarding;
- Baggage unloading/loading.

#### 4.1.7. Passenger Activity Analysis

One output of the frame differencing branch is passenger deboarding and boarding detection. Motion spikes in relevant regions indicate the collective movement of passengers between the terminal and the aircraft. By analyzing the temporal pattern of motion activity, the system can estimate when deboarding begins, when it ends, and when boarding starts later in the turnaround sequence.

#### 4.1.8. Baggage Activity Analysis

Another output of the frame differencing branch is baggage unloading and loading detection. Similar to passenger analysis, motion spikes are used to infer baggage handling activity, especially in regions where baggage carts, containers, or loaders interact with the aircraft.

#### 4.1.9. Metrics and Logs Processing

The outputs from both main branches are then forwarded to the metrics and logs processing stage. Here, the system organizes all detected events and tracking information into structured records. This includes:

- Event timestamps;
- Event durations;
- Tracked-object logs;
- Operational metrics;
- And summarized activity information.

This stage transforms low-level frame-by-frame results into higher-level analytical outputs that can be used for performance evaluation and airport turnaround monitoring.

#### 4.1.10. Final Outputs

The final stage of the workflow produces several outputs:

- Processed video containing visual annotations of detections, tracks, and inferred events;
- Gantt charts, which summarize the temporal structure of turnaround activities;
- CSV files, which store detailed logs and numerical results for later analysis.

**Figure 6:**
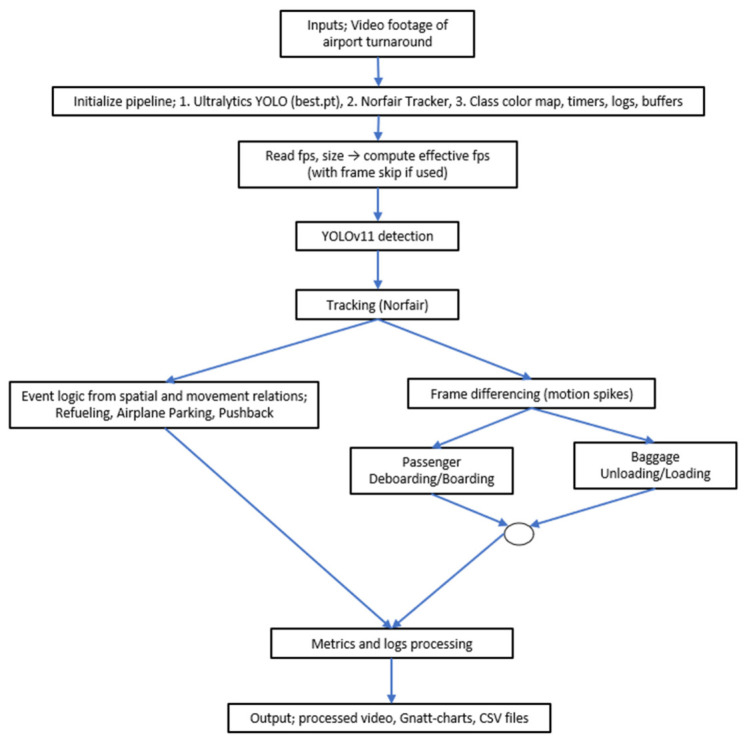
Overall workflow of the proposed computer vision-based airport turnaround monitoring pipeline. The pipeline starts from input video acquisition, followed by YOLOv11 object detection, Norfair multi-object tracking, frame differencing-based motion analysis, event logic processing, and final output generation, including annotated video, CSV logs, and Gantt chart-based turnaround activity timelines.

These outputs make the system suitable for both visual inspection and quantitative study. The processed video supports interpretability, the Gantt chart supports operational timeline analysis, and the CSV files support reproducibility and downstream statistical evaluation. All of these steps are summarized in Figure 6 below.

The modules in the proposed pipeline were selected according to the operational requirements of airport turnaround monitoring rather than as a random combination of existing tools. YOLOv11 was selected as the object detection module because the system first requires reliable frame-level identification of aircraft, aerobridge, belt loader, tractor, baggage container, fuel-related equipment, tow tug, and other turnaround objects. Norfair tracking was then used to provide temporal continuity by maintaining object identities across consecutive frames, which is necessary for estimating object presence, movement, and duration. However, object detection and tracking alone are not sufficient to identify passenger and baggage activities from a fixed long-distance airport camera view. Therefore, region-of-interest frame differencing was added to capture motion changes in operationally meaningful areas, especially the aerobridge window region for passenger movement and the belt loader roof region for baggage movement. Finally, rule-based temporal event parsing was used because airport turnaround activities follow known spatial and temporal relationships among aircraft, ground support equipment, and motion cues. This design allows the pipeline to transform object detections and motion signals into structured operational outputs, including timestamped logs and Gantt-style turnaround timelines.

### 4.2. YOLO and YOLOv11

You Only Look Once (YOLO) is a one-stage object detection framework that performs object localization and classification in a single forward pass, making it well suited to real-time vision tasks. Unlike two-stage detectors that first generate region proposals and then classify them, YOLO directly predicts bounding boxes and class probabilities from the input image, which reduces latency and enables efficient deployment in video-based applications. In practical research settings, YOLO is widely used when a balance between detection accuracy and processing speed is required [31,32].

YOLOv11 is the Ultralytics generation of the YOLO family designed to support multiple computer vision tasks, including object detection, instance segmentation, image classification, pose estimation, and oriented object detection. According to the official Ultralytics documentation, YOLOv11 is provided in multiple model scales and is intended for training, validation, inference, and export across different deployment environments. For an academic methodology section, YOLOv11 can therefore be described as a real-time deep learning detector selected for its strong detection capability, flexible deployment, and compatibility with modern training and validation workflows [33,34,35].

In this study, YOLOv11 was adopted as the core object detector to identify operational targets from video frames. The main rationale for this choice is that YOLOv11 provides a practical trade-off between computational efficiency and detection performance, which is important in airport-turnaround analysis where multiple object classes may need to be detected continuously across long video sequences. In methodology terms, YOLOv11 may be presented as the primary spatial feature extractor that outputs class labels, confidence scores, and bounding boxes for downstream temporal analysis.

### 4.3. Norfair Tracking

Norfair is a tracking-by-detection library designed to track multiple objects in video based on detections produced by an external detector. Its core idea is to receive frame-by-frame detections, associate them with existing tracked objects using a distance function, and maintain object identities over time. This makes Norfair especially suitable for pipelines in which a detector such as YOLO is responsible for spatial localization, while a dedicated tracker is responsible for temporal continuity [36,37,38,39,40].

The Norfair tracker performs data association by comparing newly detected objects with already tracked objects through a user-defined or predefined distance function. The official tracker reference explains that the *distance_threshold* determines the maximum distance allowed for a valid match, while the distance function itself defines how similarity between detections and tracked objects is measured. In practice, this mechanism allows the system to preserve object identities across consecutive frames, estimate trajectories, and support time-based event analysis.

Norfair can be described as the temporal module of the proposed pipeline. After YOLOv11 detects objects in each frame, Norfair links detections across time to form continuous tracks. This step is important because airport turnaround interpretation depends not only on whether an object appears in one frame, but also on how long it remains present, when it enters or leaves the scene, and how its position evolves over time.

### 4.4. Frame Differencing

Frame differencing is a classical motion detection technique that identifies moving regions by comparing adjacent video frames and highlighting pixels that change significantly over time. In computer vision, it is commonly used as a lightweight motion cue because it suppresses static background content and emphasizes temporal changes caused by moving objects or local activities. This makes it particularly useful when subtle motion needs to be detected in predefined regions of interest [41].

#### A New Method for Motion Target Detection by Background Subtraction and Update

More broadly, frame differencing belongs to the family of foreground extraction and motion detection methods used in video analysis. Reviews of moving object detection note that background subtraction and frame difference approaches remain important for locating motion in image sequences, especially when they are used as a first-stage signal before higher-level recognition or tracking. Their main advantage is computational simplicity, although they can be sensitive to illumination changes, dynamic backgrounds, and camera motion [42,43,44].

Let $I_{t}\left(x,y\right)$ denote the pixel intensity at spatial position (x,y) in frame t, and $I_{t-1}\;\left(x,y\right)$ denote the corresponding pixel intensity in the previous frame. The basic frame difference image $D_{t}(x,y)$ is computed as

(1) $$\;D_{t}(x,y)=|I_{t}(x,y)-I_{t-1}\;(x,y)|$$

This operation emphasizes pixels whose intensities have changed between two consecutive frames, which are likely associated with motion.

To separate moving pixels from non-moving pixels, a threshold T is typically applied as follows:

(2) $$\;M_{t}(x,y)=\left\{\begin{array}{c} 1,\;\;\;D_{t}\left(x,y\right)>T,\\ 0,\;\;\;D_{t}\left(x,y\right)\leq T, \end{array}\right.$$

where $M_{t}\left(x,y\right)$ is the resulting binary motion mask. A value of 1 indicates detected motion, whereas 0 indicates background or insignificant change.

For region-based motion analysis, the amount of motion inside a region of interest (ROI) Ω can be summarized by the average difference magnitude as follows:

(3) $$\;S_{t}=\frac{1}{|\Omega |}\sum_{(x,y)\in \Omega }D_{t}(x,y)$$

where ∣Ω∣ is the number of pixels in the ROI. A motion event may then be declared when $S_{t}$ exceeds a predefined threshold as follows:

(4) $$\;Motion\;Event\;at\;time\;t⟺S_{t}>\tau ,$$

where τ is an empirically chosen motion threshold. This ROI-based formulation is especially suitable when the objective is to monitor localized activities, such as passenger movement or baggage handling motion, rather than full-scene object motion [45,46,47].

In methodology terms, frame differencing can be introduced as a complementary temporal analysis technique that augments detector-based recognition. While YOLOv11 captures semantic object categories, frame differencing provides direct evidence of motion between consecutive frames. In airport turnaround monitoring, this is useful for identifying fine-grained operational activities, such as localized passenger movement or baggage handling motion, particularly in regions where small targets may be partially occluded or difficult to detect robustly using bounding boxes alone.

### 4.5. Performance Metrics

The performance of an object detection model is commonly evaluated using precision, recall, intersection over union (IoU), and mean average precision at an IoU threshold of 0.50 (mAP50). Ultralytics presents these as standard validation metrics for measuring classification correctness and localization quality in object detection tasks.

Let TP, FP, and FN denote the numbers of true positives, false positives, and false negatives, respectively. Precision is defined as

(5) $$Precision\;(P)=\frac{TP}{TP+FP}\;$$

which measures the proportion of predicted detections that are correct. A higher precision indicates fewer false alarms.

Recall is defined as

(6) $$Recall\;\left(R\right)=\frac{TP}{TP+FN}\;$$

which measures the proportion of actual objects that are successfully detected. A higher recall indicates fewer missed detections.

Intersection over union (IoU) quantifies the overlap between a predicted bounding box $B_{p}$ and a ground-truth bounding box $B_{g}$. It is defined as

(7) $$IoU=\frac{\left|B_{p}\cap \;B_{g}\right|}{\left|B_{p}\cup \;B_{g}\right|}\;$$

where $\left|B_{p}\cap B_{g}\right|$ is the area of overlap and $\left|B_{p}\cup B_{g}\right|$ is the area of union between the two boxes. IoU is fundamental in object detection because it determines whether a predicted box is sufficiently aligned with the corresponding annotation.

To summarize performance over varying confidence thresholds, the average precision (AP) for one class is computed as the area under the precision-recall curve as follows:

(8) $$AP=\int_{0}^{1}P\left(R\right)\;dR\;$$

where $P\left(R\right)$ represents precision as a function of recall. In practice, this integral is approximated numerically from the discrete precision–recall curve obtained during validation.

For a multi-class detection problem with C classes, mean average precision at IoU 0.50 is defined as

(9) $$mAP@50=\frac{1}{C}\sum_{c=1}^{C}{AP}_{c}^{\left(0.50\right)}$$

where ${AP}_{c}^{\left(0.50\right)}$ is the average precision of class c when a detection is counted as correct only if its IoU with the ground truth is at least 0.50. Thus, mAP50 provides an overall summary of detection quality across all classes under the commonly used IoU threshold of 0.50 [48,49,50,51,52].

## 5. Experimental Results

The experiments in this study were conducted using the dataset presented in Table 2, and the corresponding training and testing results are shown in Table 3. Training was performed using an NVIDIA L40S GPU, designed by NVIDIA Corporation (Santa Clara, CA, USA). The YOLOv11n model was trained with an image size of 640, a batch size of 16, a patience of 100, and a maximum of 3000 epochs. After training, the video processing and event extraction pipeline was also tested on a Lenovo IdeaPad Gaming 3 15IAH7 laptop, designed by Lenovo Group Limited (Beijing, China) to examine its practical usability on consumer-grade hardware. For each input video, the processing script automatically reads the original video frame rate, width, and height, and preserves the original frame size in the processed output. A frame-skipping strategy was applied with skip = 6, resulting in an effective processing frame rate of fps/6. This setting was selected to reduce computational load and support near-real-time processing, where the processing time was approximately comparable to the original video duration on the tested laptop. For example, a 30 min video required approximately 30 min to process in this setup. In addition, detected object positions were converted into a normalized 0–200 coordinate scale to support consistent spatial logging across videos.

As presented in Table 3, the overall precision, recall, and mAP50 on the test set all exceed 90%, demonstrating that the trained YOLOv11 model achieved strong overall detection performance for airport turnaround video analysis. However, class-level results also indicate that some small or visually ambiguous components, particularly the fuel tube class, require further improvement before engineering-level deployment. Although the overall detection performance was strong, the fuel tube class showed noticeably lower performance than the other classes, with a test mAP50 of 0.7033, recall of 0.5347, and precision of 0.6909. This indicates that fuel tube detection remains a limitation of the current model. The lower performance is likely related to the small and thin visual appearance of the fuel tube, partial occlusion, long-distance camera perspective, and low contrast between the fuel tube and surrounding aircraft or ground service equipment. Therefore, the current fuel tube result may not yet be sufficient for strict engineering deployment if fuel tube detection is required as an independent safety-critical output. However, in the proposed turnaround monitoring pipeline, the refueling stage is not inferred from the fuel tube alone, but also from related refueling cues such as the fuel line and fuel tanker. Future work should improve this class by increasing fuel tube samples, adding more varied annotations under different lighting and occlusion conditions, applying small-object augmentation, and testing higher-capacity YOLOv11 variants.

**Figure 7:**
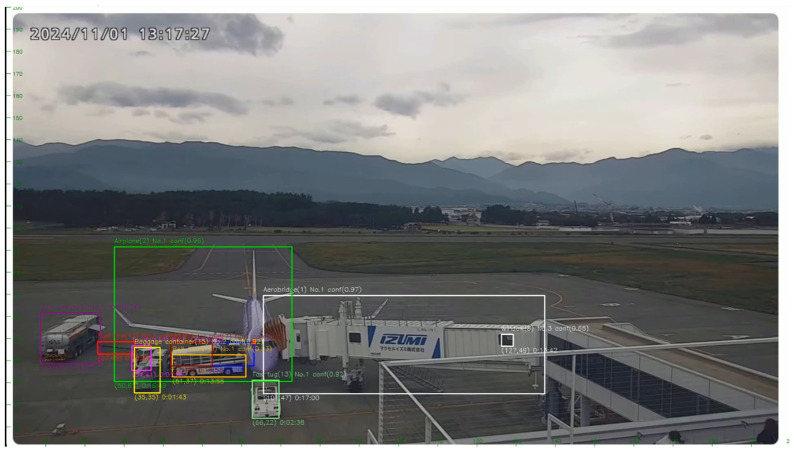
Screenshot of Airport Turnaround Video Processed by the Trained YOLOv11 Model.

Figure 7 illustrates a screenshot from the airport turnaround process, where the trained YOLOv11 model was employed to analyze the video and extract information on key operational stages, including airplane parking, passenger deboarding/boarding, and baggage unloading/loading.

**Figure 8:**
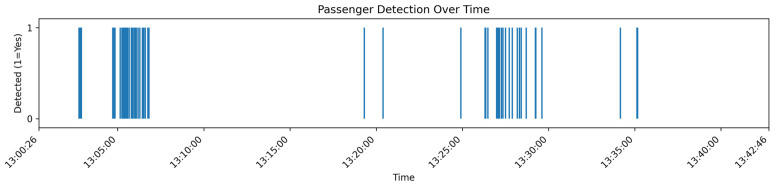
Passenger Detection During Airport Turnaround Using Frame Differencing in the Aerobridge Window Region.

Figure 8 presents the results of passenger detection during the airport turnaround process. Two distinct clusters of blue-line spikes can be observed on the left and right sides of the figure, corresponding to passenger movement detected by the proposed frame differencing approach. Specifically, the trained YOLOv11 model was first used to identify the aerobridge window region, and frame differencing was subsequently applied within this area to capture motion across consecutive frames. Whenever passenger movement occurred, the resulting pixel changes were recorded as blue-line spikes. The first cluster on the left indicates passenger deboarding, as it takes place shortly after the aircraft has parked at the stand. In contrast, the second cluster on the right corresponds to passenger boarding, occurring after a considerable time interval from the first cluster, which represents the ground preparation period before the aircraft’s next departure.

**Figure 9:**
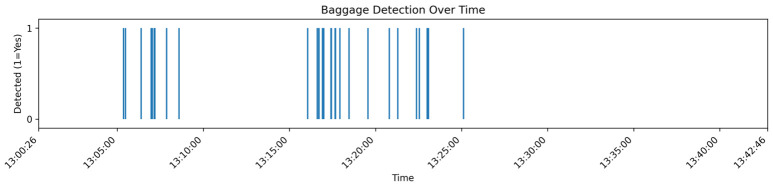
Baggage Detection During Airport Turnaround Using Frame Differencing in the Belt Loader Roof Region.

Figure 9 presents the detection of baggage movement during the airport turnaround process, based on a method similar to that used for passenger detection. The trained YOLOv11 model was first employed to detect the belt loader and define the belt loader roof region as the area of interest. Subsequently, frame differencing was applied within this region to identify motion across consecutive frames. When baggage moved through the bounding box area associated with the belt loader roof, the detected motion was recorded as blue-line spikes. The first cluster on the left represents baggage unloading, as it occurs shortly after the aircraft has parked at the stand. The second cluster represents baggage loading, which takes place after a noticeable time interval following the first cluster, indicating the aircraft preparation period before the next flight.

**Figure 10:**
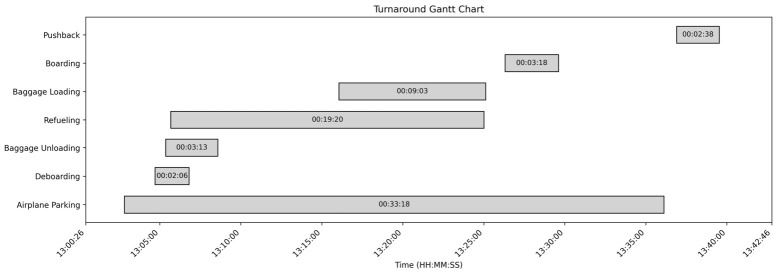
Generated Gantt Chart of Airport Turnaround Activities Based on Extracted Operational Data.

After extracting data from all equipment associated with the airport turnaround process, namely (a) tow tug, (b) aerobridge, (c) airplane, (d) baggage container, (e) belt loader, (f) belt loader roof, (g) fuel line, (h) fuel tanker, (i) fuel tube, (j) tractor, and (k) window, together with the passenger and baggage movement data obtained as described previously, a Gantt chart was generated, as presented in Figure 10. This chart visualizes the duration of each key turnaround activity, including airplane parking, deboarding, baggage unloading, refueling, baggage loading, boarding, and pushback.

## 6. Discussion and Conclusions

Airport turnaround is a complex operational process that involves multiple ground activities occurring sequentially and in parallel. These activities include aircraft parking, aerobridge connection, passenger deboarding and boarding, baggage unloading and loading, refueling, and pushback. In current airport operations, these processes are commonly monitored by human operators, ground staff, or operational records. Although these conventional approaches are useful, they can be time-consuming and may not always provide detailed and consistent timestamps for each activity. Therefore, this study proposed a computer vision-based airport turnaround monitoring pipeline to automatically detect important ground-operation objects, analyze passenger and baggage-related motion, and convert the extracted information into a structured operational timeline.

The experimental results show that the trained YOLOv11 model achieved strong object detection performance on the airport turnaround image dataset. As reported in Table 3, the overall test precision, recall, and mAP50 were 0.9609, 0.9445, and 0.9617, respectively. These results indicate that the model was able to detect most key turnaround objects with high accuracy under the dataset conditions used in this study. Large and frequently visible objects, such as the airplane, aerobridge, belt loader, belt loader roof, and fuel tanker, achieved particularly strong performance. This is important because these objects are directly associated with several major turnaround stages, including aircraft parking, aerobridge connection, baggage handling, refueling, and pushback.

However, the detection results also show that some object classes remain challenging. In particular, the fuel tube class achieved substantially lower performance than most other classes. This is likely due to its small size, thin shape, partial occlusion, low visibility, and visual similarity to surrounding aircraft components, ground equipment, shadows, and nighttime background regions. This finding highlights an important limitation of applying object detection to long-distance airport surveillance footage, where small or low-contrast objects may be difficult to detect consistently. Therefore, although the overall YOLOv11 detection performance was strong, the weaker performance of the fuel tube class indicates that the refueling-related part of the pipeline still requires further improvement.

A key contribution of this study is that the proposed system does not rely only on object detection. While YOLOv11 provides useful semantic information about visible airport objects, some important turnaround activities cannot be reliably identified from bounding boxes alone. Passenger deboarding and boarding may occur inside or near the aerobridge, where individual passengers are small, partially hidden, or not clearly visible from the camera viewpoint. Similarly, individual baggage items may be too small or unclear to be labeled and detected reliably as separate objects. To address this issue, this study incorporated frame differencing as a complementary motion-based method. By applying frame differencing within selected regions of interest, the system was able to detect motion patterns associated with passenger and baggage activities.

The passenger activity results show that motion spikes in the aerobridge window region can provide temporal evidence of passenger deboarding and boarding. The first major motion cluster appears after aircraft parking and corresponds to deboarding, while a later motion cluster corresponds to boarding. Similarly, the baggage activity results show that motion spikes in the belt loader roof region can support the interpretation of baggage unloading and baggage loading. These results suggest that localized motion analysis can help infer activity-level information, especially when individual objects are too small or partially occluded for reliable direct detection.

The integration of YOLOv11 object detection, Norfair multi-object tracking, and frame differencing therefore provides a more complete monitoring framework than using object detection alone. YOLOv11 identifies the presence and location of key turnaround objects, Norfair tracking supports temporal continuity across video frames, and frame differencing adds motion-based evidence for activities involving small or partially visible objects. By combining these components, the proposed pipeline can transform frame-level detections and motion signals into higher-level operational outputs, including event logs and a Gantt-style turnaround timeline.

The generated Gantt chart demonstrates the practical potential of the proposed approach. It summarizes key turnaround activities, including aircraft parking, deboarding, baggage unloading, refueling, baggage loading, boarding, and pushback. Such a timeline can help airport operators, ramp managers, and researchers understand when each activity starts, how long it lasts, and how different activities overlap. In future real-time or near-real-time deployment, this type of output could support delay identification, turnaround performance comparison, and improved coordination of ground resources.

Nevertheless, the findings of this study should be interpreted carefully. The current work demonstrates the feasibility of using a single-camera computer vision pipeline for airport turnaround monitoring, but it should not yet be considered a fully validated operational system. First, the dataset was collected from one airport camera source, meaning that the camera angle, airport layout, aircraft type, and ground operation pattern may not fully represent other airport environments. Second, the train, validation, and test sets were created using an image frame-level split. Since frames from the same original video may appear across different subsets, the object detection results may be more optimistic than results obtained from a strict video-level or airport-level split. Third, the motion-based frame differencing method depends on manually selected regions of interest and motion thresholds. Changes in lighting, heavy rain, reflections, shadows, camera vibration, occlusion, or nighttime illumination may introduce noise into the motion signal. Fourth, the current pipeline has not yet been fully validated using large-scale independent manual ground truth timestamps for all operational events. Therefore, the results should be regarded as a proof-of-concept demonstration of the proposed monitoring framework rather than definitive evidence of operational-level accuracy.

These limitations also indicate important directions for future research. Future work should expand the dataset to include more airports, aircraft types, stand layouts, camera viewpoints, and environmental conditions. A stricter video-level or airport-level dataset split should also be used to better evaluate generalization to unseen turnaround sequences. In addition, larger-scale manual annotation of event start and end times should be conducted to evaluate timestamp error, duration error, and event-level precision and recall. For refueling detection, future work should improve the detection of small and low-contrast objects such as fuel tubes and fuel lines, possibly by using higher-resolution imagery, additional camera viewpoints, small-object detection techniques, or multi-sensor information. More advanced temporal models, such as recurrent neural networks, transformer-based video models, or graph-based event reasoning, could also be explored to improve event recognition robustness.

In conclusion, this study presented a modified YOLO-based computer vision pipeline for airport turnaround monitoring. The proposed method combines YOLOv11 object detection, Norfair multi-object tracking, and frame differencing-based motion analysis to detect key airport ground operation objects and infer passenger and baggage-related activities from video footage. The trained YOLOv11 model achieved strong overall object-detection performance, with test precision, recall, and mAP50 all above 90%. The system was also able to generate a Gantt-style timeline summarizing major turnaround activities. These results show that the proposed approach has potential to transform airport video footage into structured operational information. However, because the study is limited by a single airport camera viewpoint, frame-level dataset splitting, environmental noise, small-object detection challenges, and limited event-level ground truth validation, the current system should be regarded as a proof-of-concept framework. Further validation and technical refinement are required before the method can be applied as a reliable operational decision-support system in real airport environments.

## Figures and Tables

**Table 1:** Lists of related research and commercial work using artificial intelligence to manage airport turnaround process.

No., Year of Publication, Citation	Method Used	Type of Monitor Device or Input Source	Accuracy of Model	Baggage Unloading/Loading, Passenger Deboarding/Boarding
1., 2008 [9],	Real-time aircraft turnaround monitoring framework (ATMS) and Turnaround Operation Monitoring Agent (TOM)	Mobile computing devices (PDAs) and wireless network technology General Packet Radio Service (GPRS)	Validated but not mentioned	Bridge attach/detach; cargo-loader attach/detach.
2., 2018 [10],	Machine learning (long short-term memory, LSTM) for prediction	Simulated data via calibrated stochastic boarding model; aggregated into a time-based “complexity” metric	Deviation reduced up to 75%; residual difference of ±20 s	Agent-based passenger boarding simulation; no baggage/bridge/loader event detection.
3., 2020 [11],	Tree-based machine learning algorithm XGBoost and interpretability framework SHAP for prediction	Historical operational airport/airline databases	Mean absolute error of 2.81 min and explained variance (R^2^) of 0.60.	Uses passenger count and cargo amount as variables to predict overall turnaround duration.
4., 2020 [12],	ConvNet-based models (AirNet) for object detection, tracking, and activity detection	Live gate cam (surveillance camera)	Aircraft type recognition’s accuracy is 100%; object detection’s mAP is 0.9514; activity detection’s median error is <6 s	Detects bridge attachment/detachment and cargo loader attachment/detachment events.
5., 2023 [13,14],	Integration of AI, predictive analytics, machine learning, computer vision, and real-time data processing	Cameras and sensors inside Safedock A-VDGS	Not publicly reported (commercial solution)	Bridge connection tracking confirmed; baggage load/unload not explicitly listed.
6., 2022 [15],	AirNet (Custom CNN) with depthwise convolution	Multiple cameras (wide field-of-view)	Average precision of object detection is approximately 97%; average precision of the AirNet is 85%	Detects GSE–aircraft interactions (bridge/cargo-loader attachment/detachment)
7., 2022 [16],	Machine learning on turnaround sub-process durations + fusion of overlaps	Historical operational turnaround data, specifically the durations of sub-processes	Classification accuracy: decision tree 76.32%, random forest 83.22%. Random forest RMSE improved 4.64 to 4.36 min	Uses 7 sub-process durations (operations record data; not computer vision)
8., 2023 [17],	Computer vision key milestone nodes (KMNs) framework (improved YOLOv5 + Kalman filtering + Hungarian association)	Fixed airport surveillance cameras	Precision up to 93.6%, recall 93.1%, mAP 94.7%, multi-object tracking accuracy (MOTA) 95.09%	Detects only in-/off-block + stairs docking/undocking (passenger access proxy); no baggage unload/load detection.
9., 2022 [18],	Deep learning computer vision (YOLOv4/YOLO-tiny/SSD) for detection; CSRT/MOSSE tracking; Haar/TextBoxes + CRNN for tail numbers	Intelligent cameras (passive system)	Haar: Precision 84%, recall 77%. TextBoxes: Precisison 91%, recall 83%. Aircraft % (correctly) identified: 80% in Layout 2	Aircraft computer vision only; no baggage/passengers activity detection.
10., 2022 [19],	Deep learning computer vision system for auto-detecting/tracking ground service actions + start/end timestamps	RGB video frame sequences (single fixed apron camera)	Precision rates over 90%” for detecting/analyzing ground services	Optical flow ROI around door/ladder and belt loader; mean flow direction/thresholds for boarding/deboarding and loading/unloading (rule-aided).
11., 2024 [20],	Improved YOLOv5 (with SPD-Conv block) + activity identification	Apron surveillance-camera video (real operational footage)	Detection average precision of all objects is >90%; whole-class mAP 98.7%, with GPU/CPU inference efficiency +55.3%/+137.1%	Recognizes GSE/door states: Bridge connected and passenger door open (pax proxy); baggage loading via belt-loader/tractor at hatch.
12., 2024 [21],	Time Transition Petri Net (TTPN) and Bayesian theorem (modeling)	Historical operational records	RMSE: 3.75 min. MAE: 3.40 min.	Focuses on time prediction of the overall process.
13., (Date not available) [22],	AI-enabled video analytics engine	Apron surveillance camera video	Not publicly reported (commercial white paper)	Baggage/cargo unloading monitoring: Detects belt loaders and baggage trucks and determines active status; passenger phases inferred from object/state cues.
14., 2024 [23],	AI model using computer vision, Pytorch, OpenCV, and CNN	Existing cameras in the terminal.	Per-event success (Phase-1) of 16 events: 63–100%	Monitors all processes of ground operations via GSE/objects and state cues.
15., (Date not available) [24],	AI-driven computer vision (real-time tracking/counting, automated sorting and reading barcode tags)	Video feeds from baggage claims and gates.	Not publicly reported (commercial solution)	Baggage/cargo: Real-time volume tracking + automated sorting/tracking. Passenger: Gate video estimates hand luggage counts vs. flight manifest (phase inference)

Note: GSE stands for Ground Support Equipment.

**Table 2:** Summary of object classes by training, validation, and test sets.

Object Classes	Dataset (1446 Images)		
	Train	Validation	Test
Tow tug	683 instances	237 instances	79 instances
Aerobridge	1012 instances	288 instances	146 instances
Airplane	1005 instances	287 instances	145 instances
Baggage container	299 instances	77 instances	52 instances
Belt loader	569 instances	145 instances	91 instances
Belt loader roof	567 instances	145 instances	90 instances
Fuel line	377 instances	67 instances	78 instances
Fuel tanker	565 instances	127 instances	106 instances
Fuel tube	253 instances	48 instances	46 instances
Tractor	297 instances	78 instances	52 instances
Window	753 instances	228 instances	118 instances
number of total images	1012 images	288 images	146 images

**Table 3:** Accuracy of the YOLOv11 model in detecting objects by class.

Object Classes	Train			Validation			Test		
	*p*	R	mAP50	*p*	R	mAP50	*p*	R	mAP50
Tow tug	0.9987	1	0.995	0.9956	1	0.9918	0.9915	0.9873	0.9949
Aerobridge	0.9993	1	0.995	0.9984	1	0.995	1	1	0.995
Airplane	0.9992	1	0.995	0.9987	0.993	0.995	0.9954	1	0.995
Baggage container	0.9979	0.9933	0.995	0.9613	0.987	0.9929	0.9914	0.9808	0.9886
Belt loader	0.9987	1	0.995	0.9918	1	0.995	0.9922	1	0.995
Belt loader roof	0.9983	1	0.995	0.9925	1	0.995	0.9864	1	0.995
Fuel line	0.9975	0.9947	0.995	0.9929	0.9701	0.994	0.9922	0.9872	0.9905
Fuel tanker	0.9986	1	0.995	0.9977	1	0.995	0.9927	1	0.995
Fuel tube	0.8413	0.8802	0.9174	0.8989	0.8542	0.9488	0.6909	0.5347	0.7033
Tractor	0.992	1	0.995	0.9713	0.9744	0.9877	0.9773	0.9423	0.9749
Window	0.999	1	0.995	0.9983	1	0.995	0.9604	0.9576	0.9519
Overall	0.9837	0.988	0.9879	0.9816	0.9799	0.9896	0.9609	0.9445	0.9617

## Data Availability

Available Datasets at [53]: https://app.roboflow.com/airport-turnaroundmy-own/airport_turnaround_nagano_real1/2 (accessed on 18 May 2026).
